# Iron Overload Mimicking Conditions Skews Bone Marrow Dendritic Cells Differentiation into MHCII^low^CD11c^+^CD11b^+^F4/80^+^ Cells

**DOI:** 10.3390/ijms21041353

**Published:** 2020-02-17

**Authors:** Giulio Verna, Marina Liso, Stefania De Santis, Manuela Dicarlo, Elisabetta Cavalcanti, Alberto Crovace, Annamaria Sila, Pietro Campiglia, Angelo Santino, Antonio Lippolis, Grazia Serino, Alessio Fasano, Marcello Chieppa

**Affiliations:** 1 National Institute of Gastroenterology “S. de Bellis”, Research Hospital, Castellana Grotte, 70013 Bari, Italy; g.verna@ebris.eu (G.V.); marinaliso@libero.it (M.L.); manueladicarlo@alice.it (M.D.); elisabetta.cavalcanti@irccsdebellis.it (E.C.); alberto.crovace@libero.it (A.C.); a.sila@outlook.it (A.S.); antonio.lippolis@irccsdebellis.it (A.L.); graziaserino@virgilio.it (G.S.); 2 Department of Immunology and Cell Biology, European Biomedical Research Institute of Salerno (EBRIS), 84125 Salerno, Italy; pcampiglia@unisa.it (P.C.); afasano@mgh.harvard.edu (A.F.); 3 Department of Pharmacy, University of Salerno, 84084 Fisciano, Italy; s-desantis@live.it; 4 Department of Pharmacy-Drug Science, University of Bari Aldo Moro, 70126 Bari, Italy; 5 Unit of Lecce, Institute of Sciences of Food Production C.N.R., via Monteroni, 73100 Lecce, Italy; angelo.santino1317@gmail.com; 6 Harvard Medical School Division of Pediatric Gastroenterology and Nutrition and Mucosal Immunology and Biology Research Center, Massachusetts General Hospital for Children, Boston, MA 02114, USA

**Keywords:** dendritic cells, inflammation, iron overload, bone marrow

## Abstract

Iron overload is an undesired effect of frequent blood transfusions or genetic diseases. Myelodysplastic syndrome (MDS) patients become transfusion dependent, but due to the combination of ineffective haematopoiesis and repeated blood transfusions they are often subject to iron overload. In this study, we demonstrate that iron-overload mimicking condition alters bone marrow progenitor differentiation towards dendritic cells (DCs). Cells cultured in iron-enriched culture medium for seven days fail to differentiate into conventional CD11c^+^MHCII^hi^ DCs and fail to efficiently respond to LPS (Lipopolysaccharides). Cells appear smaller than control DCs but vital and able to perform FITC-dextran (Fluorescein isothiocyanate-dextran) endocytosis. At molecular level, cells cultured in iron-enriched conditions show increased *ARG1* and *PU.1*, and decreased *IRF8* expression.

## 1. Introduction

Iron is a crucial micronutrient for humans as it is fundamental for the development of red blood cells and the support of efficient immune function. Iron homeostasis is finely regulated to avoid undesired iron loss during healthy periods. Lost iron is promptly replaced from dietary products in fragile populations (especially in low-income countries). Iron deficiency is very common and is the main cause of anemia [1]. Indeed, iron is incorporated into hemoglobin, which is the metalloprotein responsible for oxygen transport that accounts for about 96% of the erythrocytes dry content. Several mechanisms take place to recycle iron ions from degraded proteins and cofactors. Most of them rely on immune cells, particularly spleen resident macrophages that recycle iron from the hemoglobin of old red blood cells [2].

When iron is released into the circulation, it binds to transferrin, a plasma glycoprotein, that is able to reversibly bind iron with high affinity. In healthy conditions, transferrin saturation is approximately 30%, but when circulating iron saturates transferrin’s binding capacity, non–transferrin-bound iron (NTBI) is produced [3].

Ineffective hematopoiesis leading to peripheral blood cytopenia is often caused by myeloid malignancies like myelodysplastic syndrome (MDS) [4] but also by different forms of anemia and β-thalassemia. Patients affected by anemia become transfusion dependent, but due to the combination of ineffective hematopoiesis and repeated blood transfusions, they are often subject to iron overload that promotes iron-induced toxicity due to the production of reactive oxygen species (ROS) [5,6]. Iron excess can be deposited in the liver, heart, spleen, pancreas, bone marrow, and other tissues, resulting in tissue damage and fibrosis [7,8]. Xin et al. recently demonstrated that iron overload impairs erythroid maturation in a model of MDS mice [9].

In the present study, we aimed to explore the effects on BMDCs (bone-marrow derived dendritic cells) differentiation when they are cultured in iron-overload mimicking conditions. Surprisingly, when BMDCs were exposed to iron-overload mimicking conditions for 7 days, a great majority of cells differentiate into MHCII^low^CD11c^+^CD11b^+^F4/80^+^ cells. Under this culture condition, cells remain alive and able to respond to LPS-induced (lipopolysaccharides) upregulation of the co-stimulatory surface proteins CD80 and CD86, but their ability to release inflammatory cytokines is reduced.

## 2. Results

### 2.1. Iron-Enriched Media Improves DCs TNFα (Tumor necrosis factor α) Secretion Ability in Response to LPS

During DCs maturation, iron uptake is important to support energy production, respiration, and metabolism. For this reason, we compared DCs response to LPS in conventional or iron-enriched media. Briefly, DCs were cultured in conventional medium with GM-CSF (Granulocyte-monocyte colony stimulating factor) and IL-4 (Interleukin) for seven days. Immature DCs were harvested and plated in conventional or iron-enriched media before LPS exposure at day seven. The supernatants were collected 24 h later.

**Figure 1:**
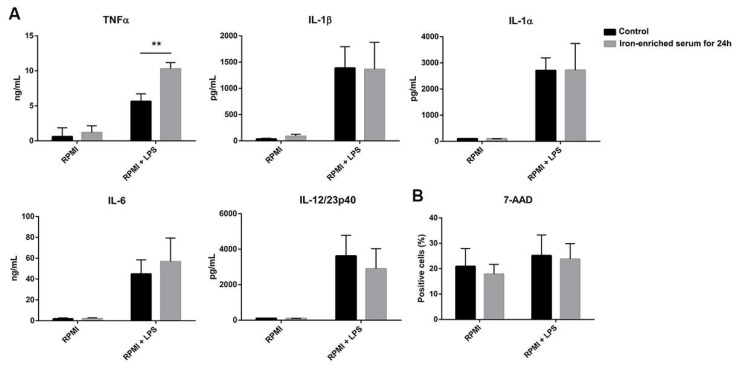
Iron added to culture medium increases the secretion of TNFα by dendritic cells (DCs) exposed for 24 h to LPS (Lipopolysaccharides) (**A**). No difference in cell vitality is observed between the two treatments (**B**) ** *p* < 0.01.

DCs cultured with iron-enriched medium for the last 24 h and activated with LPS consistently increased the secretion of the inflammatory cytokine TNFα, while IL-1α, IL-1β, IL-6, and IL-12/23p40 were not affected (Figure 1A). We did not observe any variation in cell viability too (Figure 1B).

### 2.2. DCs Reduce Inflammatory Cytokine Secretion If Cultured in Iron Overload Mimicking Conditions

Next, we evaluated whether culturing DCs in iron-enriched medium for seven days could improve the secretion of inflammatory cytokines. Briefly, DCs were cultured in conventional or iron-enriched media with GM-CSF and IL-4 for seven days. Cells were harvested, counted, and plated before LPS exposure at day seven. The supernatants were collected 24 h later and cytokine content was analyzed.

**Figure 2:**
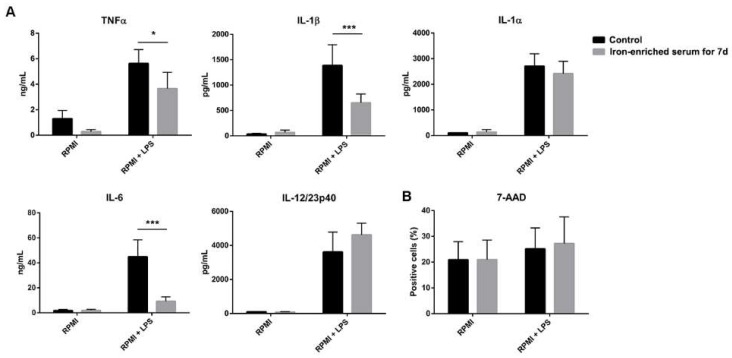
Iron added to differentiating BMDCs (Bone-marrow derived dendritic cells) blocks their differentiation, decreasing the secretion of TNFα, IL-6 (Interleukin) and IL-1β after 24 h of LPS stimulation (**A**). 7-AAD (7-Aminoactinomycin D) staining shows that cells are alive when grown in iron-enriched medium (**B**). * *p* < 0.05 *** *p* < 0.001.

Surprisingly, cells that grew for seven days in iron-enriched medium significantly reduced inflammatory cytokines (TNFα, IL-1β, and IL-6) secretion in response to LPS, while IL-1α and IL-12/23p40 secretions were not affected (Figure 2A).

The excess of labile iron has been reported to be detrimental for cell survival. Therefore, we investigated if iron-overload mimicking conditions might have decreased the vitality of DCs. Figure 2B shows that cells viability was not reduced in iron-enriched medium. When exposed to LPS, cell mortality slightly increased both in conventional culture and iron-enriched conditions (Figure 2B). In light of these results, we could exclude the hypothesis that the observed reduction of inflammatory cytokine secretion was the results of a reduction in cell number.

### 2.3. Bone Marrow Derived Cells Cultured in Iron Overload Mimicking Conditions are Significantly Smaller

**Figure 3:**
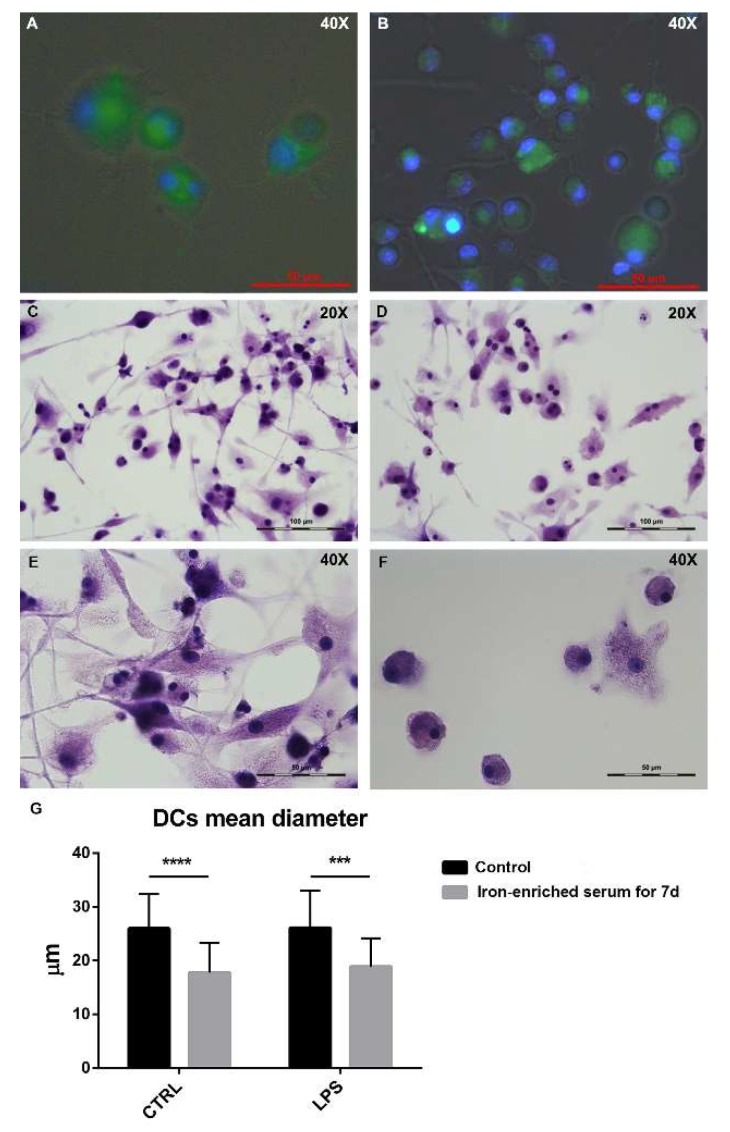
Fluorescence staining of BMDCs administered with FITC-dextran (Fluorescein isothiocyanate-dextran), control cells stimulated with LPS for 24 h (**A**) and cells grown in iron-enriched medium for seven days stimulated with LPS for 24 h (**B**). Hematoxylin staining for control cells stimulated with LPS for 24 h (**C**,**E**) and cells grown in iron-enriched medium for seven days and stimulated with LPS for 24 h (**D**,**F**) shows their different size and morphology. Bar plot shows a significant difference in DCs size (**G**). Images were taken with a 20× objective and a 40× immersion objective. *** *p* < 0.001 **** *p* < 0.0001.

BMDCs were cultured in glass bottom microwell dishes to allow cell imaging. Conventional or iron-enriched culture media were used during DCs differentiation. At day seven, cells were treated with LPS and 24 h and later they were imaged. When FITC-dextran (Fluorescein isothiocyanate-dextran) was added to the culture medium (Figure 3A,B), DCs demonstrated endocytic abilities both in conventional and iron-enriched media as shown by the green fluorescence accumulated in the cytoplasm. Hematoxylin staining highlighted the different morphology of the cells grown in iron-enriched conditions for seven days and stimulated with LPS compared to control cells stimulated with LPS (Figure 3C–F). In particular, DCs cultured in iron-overload mimicking conditions appear significantly smaller (Figure 3G). No sign of increased cell death was detectable in cells grown in iron-overload mimicking conditions.

### 2.4. Iron Overload Mimicking Conditions Skews DCs Maturation towards CD11c^+^ CD11b^+^ F4/80^+^ Cells

In light with the results obtained measuring the cytokine production, we aimed to characterize the phenotype of BMDCs cultured in iron overload conditions for seven days.

**Figure 4:**
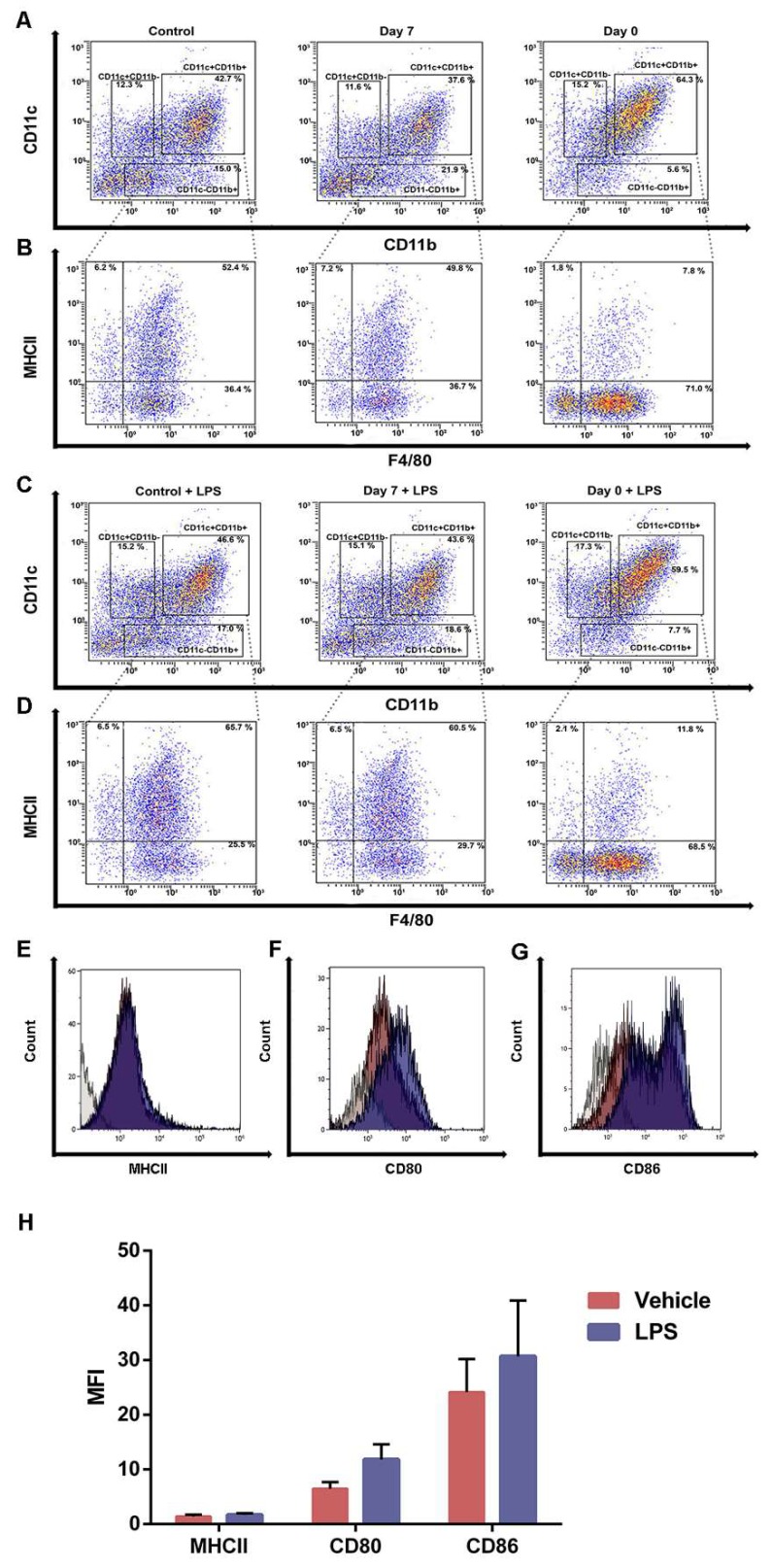
Iron blocks BMDCs maturation and generates CD11c^+^CD11b^+^F4/80^+^MHCII^low^ cells. Representative density plots and histograms of BMDCs cultured in iron-overload condition for seven days or for 24 h (**A**,**B**). BMDCs cultured in iron-overload mimicking condition for seven days or for 24 h were exposed to LPS for 24 h (**C**,**D**). Cells cultured in iron-overload mimicking condition for seven days were treated with vehicle or LPS and analyzed by FACS 24 h later (**E**–**H**). Histograms represent MHCII (**E**), CD80 (**F**), and CD86 (**G**) CD11c^+^CD11b^+^F4/80^+^MHCII^low^ cells surface expression. Bar plot showing the (Mean fluorescence index) MFI of MHCII, CD80, and CD86 (**H**).

**Figure 5:**
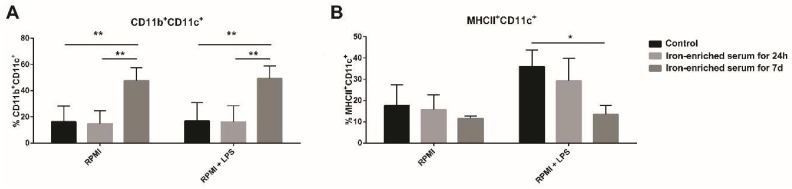
Diagrams representing the percentages of CD11c+CD11b+ cells (**A**) and CD11c+MHCII+ cells 24 h after LPS exposure (**B**). * *p* < 0.05 ** *p* < 0.01.

Bone marrow (BM) progenitors were cultured in conventional media (RPMI) or iron-enriched medium for seven days or 24 h. Cells were collected at day eight and analyzed by FACS (Fluorescence activated cell sorting). It was immediately clear that there was a substantial enrichment in the CD11b^+^CD11c^+^ population in cells exposed to iron-enriched medium for seven days (Figure 4A and Figure 5A). Gating on this population, we noticed that most of the CD11b^+^CD11c^+^ cells expressed F4/80 in iron-overload mimicking conditions (Figure 4B). Furthermore, cells cultured for seven days in iron-enriched medium dramatically reduced MHCII expression (Figure 4B and Figure 5B). These atypical DCs expressed the costimulatory molecules CD80 and CD86 (71.9% ± 4.2 and 58.0% ± 15.8, respectively, Figure 4F–G) and were GR1 and CD45R negative.

We then exposed cells grown in both control and iron-enriched medium to LPS. LPS was unable to affect the CD11c^+^CD11b^+^ population percentage in cells grown in iron-enriched medium for seven days (Figure 4C and Figure 5A). The percentage of MHCII^+^ cells increased both in control and when iron is given for 24 h (Figure 4D and Figure 5B). Indeed, the MFI value raises from 7.4 ± 0.6 to 9.8 ± 0.5 both in control and when iron is given for 24 h. Cells cultured in iron-enriched medium for seven days were not able to upregulate MHCII expression following LPS exposure like control cells (MFI: 1.4 ± 0.5 versus 1.7 ± 0.4, Figure 4D–H and Figure 5B). On the contrary, following LPS exposure, cells grown in iron-enriched medium for seven days upregulated efficiently the expression of the co-stimulatory molecules CD80 and CD86 (92.5% ± 4.2 and 81.3% ± 14.4, respectively, Figure 4F,G). Furthermore, the MFI values increase from 6.5 ± 1.7 to 11.8 ± 3.9 for CD80 and from 24.1 ± 8.5 to 30.7 ± 14.4 for CD86 (Figure 4H).

### 2.5. Bone Marrow Derived Cells Cultured in Iron-Enriched Medium for 7 Days Express TLR4 but Poorly Respond to LPS at Molecular Level

**Figure 6:**
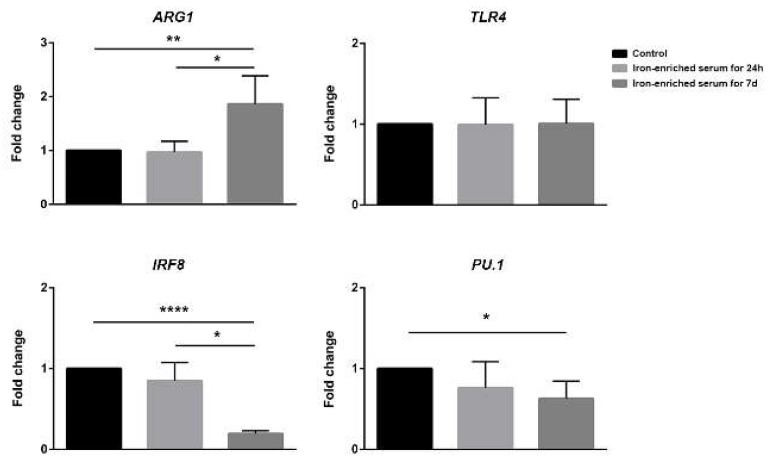
Gene expression analysis of *ARG1, TLR4*, *IRF8,* and *PU.1* in control, iron-enriched medium for 24 h or seven days cells. mRNA was isolated 6 h post LPS exposure. * *p* < 0.05, ** *p* < 0.01, **** *p* < 0.0001.

We purified the mRNA from cells cultured as previously described and analyzed the expression levels of crucial genes involved in DCs development and response to LPS. DCs cultured in conventional medium resulted IRF8^+^, PU.1^+^, TLR4^+++^, and ARG1^+^. Iron administration for 24 h did not significantly alter the expression levels of these genes if compared to control medium. Despite the reduced response to LPS, when iron-enriched medium was administered for seven days the expression of *TLR4* was not different if compared to conventional medium. On the other hand, *IRF8* and *PU.1* expression was significantly lower than the one detected in cells cultured in conventional medium, while the expression of *ARG1* was increased (Figure 6).

## 3. Discussion

Iron overload is a serious complication that affects patients with MDS and β-thalassemia. In order to overcome this phenomenon, medical treatments employ iron chelating agents that drastically increase survival rates due to reduced risks of liver and spleen dysfunction consequent to iron accumulation in the cells and progressive sideronecrosis [10]. In healthy patients, iron potential toxicity is controlled by transferrin-iron complexes. However, once transferrin is saturated non-transferrin-bound iron (NTBI) starts circulating in the blood.

Murine models of iron overload, that include KO (Knock-out) mice (*Hfe*-KO, *Hjv*-KO, *Tfr2*-KO) [11,12,13,14] and iron dextran intraperitoneal administration [14], were used to better investigate the effects that iron overload has on organs and cells. One of the most intriguing effects was reported with delayed hematopoietic reconstitution after bone marrow transplantation from healthy donors to iron-overload recipients [15]. Similarly, in patients undergoing hematopoietic stem cell transplantation, iron overload is associated with reduced survival mainly due to defects in infection containment [16].

With this study we wanted to better investigate the effects of iron overload on BMDCs differentiation in vitro. Fully differentiated DCs are potent cytokine producers and antigen presenting cells [17], they are also able to respond to elevated iron concentrations in their environment by increasing the secretion of TNFα, thus demonstrating the important role of iron in the onset of immune responses against pathogens.

Contrary to what we expected, by differentiating bone marrow progenitors under iron-overload mimicking conditions, we obtained functionally and phenotypically different cells to DCs cultured in conventional medium. We first noticed that following LPS exposure to cells grown in iron-overload mimicking condition we were not able to measure the same but reduced secreted amounts of the inflammatory cytokines IL-1β, IL-6, and TNFα as in DCs grown in conventional medium. Despite this, such suppression was not observed for IL-12/23p40 and IL-1α secretion. Intriguingly, IL-12p40 expression was observed in literature to be elevated in undifferentiated macrophages [18]. By qPCR (Quantitative polymerase chain reaction) we compared the expression of *TLR4* and detected no differences between the expression levels between cells grown in conventional medium and iron-enriched one, indicating that reduced cytokine secretion was not a side effect of reduced ability to respond to LPS.

Iron-overload did not increase cell death but a marked change in cell phenotype and morphology was observed. By FACS analysis, we detected a significant reduction in canonical CD11c^+^MHCII^hi^ cells. Following seven days of culture in iron-enriched medium, BMDCs became MHCII^low^CD11b^+^CD11c^+^F4/80^+^ cells. LPS exposure induced the expression of MHCII in a limited percentage of cells grown in iron-overload mimicking conditions, showing only a little increase in comparison to the same cells unexposed to LPS. Differently from MHCII, co-stimulatory molecules CD80 and CD86 were expressed by these cells and their expression was upregulated following LPS exposure. It is known that DCs cultured from BM progenitors in presence of GM-CSF are of heterogenous phenotype representing different cells subsets [19]. It is possible that iron-overload mimicking conditions suppress the differentiation of CD11c^+^MHCII^hi^ DCs favoring alternative cell differentiation. However, it is unknown whether the hematopoietic progenitors of CD11c^+^MHCII^hi^ cells are susceptible to iron-overload conditions or if iron abundance favors the expansion of iron-recycling cells. It is possible that inorganic iron, like heme, drives the expansion of macrophage-like cells resembling splenic red pulp macrophages as demonstrated for monocytes treated with heme by Murphy et al. [20].

Microscopic imaging clearly shows alive cells, confirming what we observe with the 7-AAD staining. Cells grown in iron-overload mimicking conditions, as well as their control counterparts, possess phagocytic ability suggesting a role similar to iron-scavenger monocytes previously reported in literature [21]. These cells are capable of phagocytize external particles even in iron-rich environment. Morphologically, these cells appear smaller and possess less dendrites compared to control DCs. The reduced cytoplasmic volume may partially explain the reduced secretory and antigen presenting ability.

This unique phenotype, together with the cell morphology, suggests that under iron-overload conditions, BM progenitors skew their differentiation towards endocytic cells with limited inflammatory abilities. This may represent a defense mechanism against undesired chronic inflammation resulting from the excess of circulating labile iron.

In line with our speculation, at a molecular level, iron-overload mimicking conditions favor the expression of *ARG1*, an enzyme mostly associated with monocyte and macrophages that is downregulated during inflammation. Since DCs differentiation is mainly driven by high levels of *PU.1* and *IRF8* [22], we also studied these two genes, detecting a decrease in their expression in cells exposed to iron-overload mimicking conditions. This result suggests us that BM progenitors exposed to high iron concentrations fail to differentiate into conventional DCs and become alternative CD11c^+^CD11b^+^MHCII^low^ cells. Once again, iron-overload cultured cells appear less differentiated and inflammatory incompetent. We realize that this may be just the first step for the understanding of the axis between circulating iron concentration and DCs differentiation, nonetheless, the present data represent the starting point for future studies in this complex field of investigation. Furthermore, we noticed that the administration of the iron-chelating polyphenol quercetin during DCs differentiation (at day five of culture) partially recovered the percentage of CD11c^+^CD11b^+^MHCII^high^ cells.

These data provide a new point of view to explain HSCT (hematopoietic stem cell transplantation) defects in patients with elevated serum iron. In these patients, bone marrow cells may undergo a defective differentiation process that is not able to support the correct immune surveillance against pathogens, thus favoring life threatening infections.

In conclusion, iron homeostasis is crucial for several aspects of the human health, including the immune response. Changing iron bioavailability may bend the balance between inflammation and tolerance, thus more needs to be clarified to design precision medicine strategies that involve iron and its metabolism.

## 4. Materials and Methods 

### 4.1. Generation and Culture of Murine BMDCs

BMDCs were obtained from wild-type mice. Six- to eight-week-old mice were sacrificed and their tibiae and femurs were flushed with 0.5mM EDTA (Thermo Fisher Scientific, MA, USA). Red blood cells were lysed with an ACK buffer (Thermo Fisher Scientific, MA, USA). The single cell suspension was plated in 10 mL dishes at the concentration of 1 × 10^6^ cells/mL in RPMI 1640 medium (Thermo Fisher Scientific, MA, USA) supplemented with 10% heat-inactivated fetal bovine serum (FBS, Thermo Fisher Scientific, MA, USA) or 10% heat-inactivated iron-enriched serum (Thermo Fisher Scientific, MA, USA), 100 U/mL penicillin/streptomycin (Thermo Fisher Scientific, MA, USA), 25ng/mL mGM-CSF (Miltenyi Biotec, Bergisch Gladbach, Germany), 25ng/mL mIL-4 (Miltenyi Biotec, Bergisch Gladbach, Germany) as previously done [23,24], and cultured at 37 °C in a humidified 5% CO_2_ atmosphere. Five days after the isolation, all non-adherent cells were gently harvested and plated on a 24-well culture plate at the concentration of 1 × 10^6^ cells/mL, new growth factors were added to the culture medium too. Iron-enriched medium was used to grow cells for seven days or added to the cell culture for 24 h. Either way, cells were terminally differentiated with 1µg/mL of Salmonella Typhimurium LPS (Sigma-Aldrich, St. Louis, MO, USA) on day seven. Six hours later, cells were harvested for mRNA analysis using TRIzol^®^ (Thermo Fisher Scientific, MA, USA) while 24 h later supernatants were collected. Our studies were conducted in accordance with national and international guidelines and approved by the authors’ institutional review board (Organism for Animal Wellbeing – OPBA). All animal experiments were carried out in accordance with Directive 86/609 EEC enforced by Italian D.L. n. 116/1992 and approved by the Committee on the Ethics of Animal Experiments of Ministero della Salute–Direzione Generale Sanità Animale (Prot. 768/2015-PR, 27 July 2015) and the official RBM veterinarian. Animals were immediately sacrificed if found to be in severe distress during the experimental period to avoid undue suffering.

### 4.2. Enzyme-Linked Immunosorbent Assay (ELISA)

Cell supernatants were analyzed for IL-12/23p40, IL-6, TNFα, IL-1β, and IL-1α release in duplicate for three consecutive and independent experiments, using ELISA kits (RD Systems, Minneapolis, MN, USA) following the manufacturer’s instructions.

### 4.3. RNA Extraction and qPCR Analysis

Total RNA was isolated from cells treated with all the aforementioned conditions using TRIzol^®^ (Thermo Fisher Scientific, MA, USA), according to the manufacturer’s instructions. Total RNA (1 µg) was reverse transcribed using an iScript cDNA Synthesis kit (Biorad, CA, USA) with random primers for cDNA synthesis. Gene expression was assessed using the following primers: *GAPDH* Mm99999915_g1 (Thermo Fisher Scientific, MA, USA), *PU.1/SPI1* Mm00488140_m1 (Thermo Fisher Scientific, MA, USA), *IRF8* Mm0042567_m1 (Thermo Fisher Scientific, MA, USA), *TLR4* Mm00445273_m1 (Thermo Fisher Scientific, MA, USA), and *ARG1* Mm00475988_m1 (Thermo Fisher Scientific, MA, USA). Real-time analysis was performed on a CFX96 System (Biorad, CA, USA) and relative expression calculated using the ΔΔCt method. At least three different experiments were performed.

### 4.4. Fluorescence Microscopy

BMDCs were cultured into 35 mm Glass Bottom Microwell Dishes (MatTek corporation, Ashland, MA, USA) and stimulated with LPS for 24 h. FITC-dextran MW 4000 (Sigma-Aldrich, St. Louis, MO, USA) was administered in the culture dish for 2 h, prior to image acquisition. Cell nucleus was stained with DAPI (Thermo Fisher Scientific, MA, USA). Images were acquired with Leica DM6000B Microscope (Leica, Wetzlar, Germany).

### 4.5. Microscopy

BMDCs were cultured into 35 mm Glass Bottom Microwell Dishes (MatTek corporation, Ashland, MA, USA) and stimulated with LPS for 24 h. Cells were fixed with two drops of PFA 4% (Sigma-Aldrich, St. Louis, MO, USA) for 2 min and then stained with two drops of hematoxylin (Diapath, Martinengo, Italy) for 4 min. The dishes were washed with dH_2_O and acquired with Leica DM6000B Microscope (Leica, Wetzlar, Germany).

### 4.6. Cytofluorimetric Analysis

Next, 24 h after LPS stimulation, BMDCs were detached from the plates with DPBS 1× (Gibco, MA, USA) + 0.5mM EDTA (Thermo Fisher Scientific, MA, USA). Cells were then washed with DPBS 1× + 0.5% bovine serum albumin (BSA, Sigma-Aldrich, St. Louis, MO, USA) and labelled with CD11b VioBrightFITC (Miltenyi Biotec, Bergisch Gladbach, Germany), MHCII PE (Miltenyi Biotec, Bergisch Gladbach, Germany), CD11c PECy5 (Miltenyi Biotec, Bergisch Gladbach, Germany), F4/80 APC (Miltenyi Biotec, Bergisch Gladbach, Germany), CD80 FITC (Miltenyi Biotec, Bergisch Gladbach, Germany), CD86 FITC (Miltenyi Biotec, Bergisch Gladbach, Germany), and 7-AAD PECy5 (Miltenyi Biotec, Bergisch Gladbach, Germany). Flow Cytometer data analysis was performed using NAVIOS software (Beckman Coulter, Brea, CA, USA), with at least three experiments performed. Flow cytometer analysis was performed using Kaluza Software 1.5 (Beckman Coulter, Brea, CA, USA).

### 4.7. Statistical Analysis

Statistical analysis was performed using GraphPad Prism 6 software (GraphPad Software, San Diego, CA, USA). All data were expressed as means ± SEM of data obtained from at least three independent experiments. We evaluated statistical significance with the two-way ANOVA test. Results were considered statistically significant at *p* < 0.05.

## Abbreviations

BMDCs	Bone marrow derived dendritic cells
DCs	Dendritic cells
HSCT	Haematopoietic stem cell transplantation
LPS	Lipopolysaccharides
MDS	Myelodisplastic Syndrome
NTBI	Non-transferrin bound iron
ROS	Reactive oxygen species
BM	Bone marrow
MFI	Mean fluorescence index
